# Supplementary material for: Microbial Degradation of Plastic in Aqueous Solutions Demonstrated by CO2 Evolution and Quantification
Source: Int J Mol Sci. 2020 Feb 11;21(4):1176. doi: 10.3390/ijms21041176 (PMC7072786; doi:10.3390/ijms21041176)
Supplement: Supplementary file 1 [file ijms-21-01176-s001.pdf]

International Journal of
Molecular Sciences

MDPI

Supplementary material

Microbial Degradation of Plastic in Aqueous Solutions Demonstrated by CO_2 Evolution and Quantification

Table S1. Assessment of reported methods for biochemical or cell staining approaches required to quantify microbial growth on plastic

Method	Advantages	Disadvantages	References
**Biochemical and spectroscopic methods:**			
ATP/ADP	High throughput; good for detecting cell viability	Cells must be detached from polymer; Potential polymer interference	[20]
Optical density at 600nm	High throughput	Measurement of turbidity which may be influenced by microplastics; cells must be detached from plastic and in suspension	[29]
Resazurin (AlamarBlue®)	High throughput; good for detecting cell viability and integrity	Chemistry of degradation may affect the assay; cells must be detached from plastic and in suspension	[22,30]
**Cell counting methods:**			
Colony forming units	Detects viable cells only; can be useful if culturing a variety of cells on the plastic	Must detach cells from plastic; heavy demand on consumables; slow process	[23]
Live/dead assay	Good to distinguish live cells and for quantification	Lengthy, technical; requires separate live and dead internal technical controls	[23]

[20] Fontanella, S.; Bonhomme, S.; Koutny, M.; Husarova, L.; Brusson, J.-M.; Courdavault, J.-P.; Pitteri, S.; Samuel, G.; Pichon, G.; Lemaire, J.; et al. Comparison of the biodegradability of various polyethylene films containing pro-oxidant additives. *Polymer Degradation and Stability* **2010**, *95*, 1011–1021.

[22] Beckloff, N.; Laube, D.; Castro, T.; Furgang, D.; Park, S.; Perlin, D.; Clements, D.; Tang, H.; Scott, R.W.; Tew, G.N.; et al. Activity of an antimicrobial peptide mimetic against planktonic and biofilm cultures of oral pathogens. *Antimicrobial Agents and Chemotherapy* **2007**, *51*, 4125–4132.

[23] Yang, J.; Yang, Y.; Wu, W.-M.; Zhao, J.; Jiang, L. Evidence of polyethylene biodegradation by bacterial strains from the guts of plastic-eating waxworms. *Environmental Science & Technology* **2014**, *48*, 13776–13784.

[29] Ali, G.S.; Reddy, A.S.N. Inhibition of fungal and bacterial plant pathogens by synthetic peptides: in vitro growth inhibition, interaction between peptides and inhibition of disease progression. *Molecular plant-microbe interactions* **2000**, *13*, 847–859.

[30] Jacks, S.; Giguère, S.; Crawford, P.C.; Castleman, W.L. Experimental infection of neonatal foals with Rhodococcus equi triggers adult-like gamma interferon induction. *Clinical and Vaccine Immunology* **2007**, *14*, 669–677.

Table S2. A summary of the molecular weight changes of LDPE and oxo-LDPE after UV irradiation and the effect on the rate of CO_2 evolution after 35 days.

Plastic	UV irradiation time (hours)	Molecular weight (kDa)	CO_2 evolved after 35 days (μmol L^{-1})*
LDPE	0	96.2	6.5 ± 1.1
LDPE	450	21.8	68.1 ± 3.5
LDPE	758	8.14	207.9 ± 10.5
LDPE	900	5.10	279.0 ± 14.1
Oxo-LDPE	0	96.2	13.0 ± 6.1
Oxo-LDPE	450	2.60	582.1 ±33.4
Oxo-LDPE	758	2.36	400.2 ± 6.4
Oxo-LDPE	900	2.22	339 ± 12.7

*± Standard error

Table S3. A description of the plastics used in this study.

Plastic	Polymer
LDPE	100% low density polyethylene
Oxo-LDPE	low density polyethylene + 1% DG12-08; Gifted from Symphony Environmental Ltd.
Compostable	Starch based compostable; off the shelf
Bioplastic	Film, compostable duplex laminate

Table S4. A complete description of the media preparation for *R. rhodochrous.* Liquid media was prepared fresh by adding 50 mL of additive to 950 mL minimal media to make up 1 L.

Chemical	Mass
**Minimal media:**	
di-sodium phosphate dodecahydrate anhydrous (Na_2HPO_4)	1.5 g
Potassium di-hydrogen phosphate anhydrous (KH_2PO_4)	1.8 g
Sodium chloride (NaCl)	0.5 g
Dissolved to a final volume of 950 mL in dH_2O (15 ΩM) and sterilized at 121 °C for 15 minutes.	
**Additive:**	
Magnesium sulfate heptahydrate ($MgSO_4.7H_2O$)	0.02 g
Ammonium iron (II) sulfate hexahydrate ($(NH_4)_2Fe(SO_4)_2.6H_2O$)	0.03 g
Calcium chloride hexahydrate ($CaCl_2.6H_2O$)	0.015 g
Ammonium chloride (NH_4Cl)	0.3 g
Trace element solution	10 μL
Dissolved to a final volume of 50 mL in dH_2O (15 ΩM) and filter sterilized through a 0.2 μm filter.	
**Trace element solution:**	
Manganese sulfate tetrahydrate ($MnSO_4.4H_2O$)	0.059 g
Boric acid (H_3BO_3)	0.029 g
Zinc sulfate heptahydrate ($ZnSO_4.7H_2O$)	0.022 g
Sodium molybdate dihydrate ($Na_2MoO_4.2H_2O$)	2.35 g
Cobalt nitrate ($Co(NO_3)_2$)	0.02 mg
Copper sulfate ($CuSO_4$)	0.02 mg
Dissolved to a final volume of 500 mL in dH_2O (15 ΩM) and filter sterilized through a 0.2 μm filter.	

Table S5. A complete description of the media (ONR7A) preparation for *A. borkumensis*. The solutions were prepared and autoclaved separately and were combined, once cooled, to prepare 1 L of culture media.

Chemical	Mass
**Solution 1:**	
Sodium chloride (NaCl)	22.79 g
Sodium sulfate (Na_2SO_4)	3.98 g
Potassium chloride (KCl)	0.72 g
Sodium bromide (NaBr)	83.0 mg
Sodium carbonate ($NaHCO_3$)	31.0 mg
Boric acid (H_3BO_3)	27.0 mg
Sodium fluoride (NaF)	2.6 mg
Ammonium chloride (NH_4Cl)	0.27 g
Di-sodium phosphate dodecahydrate dihydrate ($Na_2HPO_4.2H_2O$)	47.0 mg
TAPSO	1.3 g
Dissolved in ddH_2O (18.2 ΩM), the pH adjusted to pH 7.6 with NaOH and the final volume adjusted to 500 mL.	
**Solution 2:**	
Magnesium chloride hexahydrate ($MgCl_2.6H_2O$)	11.18 g
Calcium chloride dihydrate ($CaCl_2.2H_2O$)	1.46 g
Strontium chloride hexahydrate ($SrCl_2.6H_2O$)	24.0 mg
Dissolved to a final volume of 450 mL in ddH_2O (18.2 ΩM).	
**Solution 3:**	
Ferrous chloride tetrahydrate ($FeCl_2.4H_2O$)	2.0 mg
Dissolved to a final volume of 50 mL in ddH_2O (18.2 ΩM).	

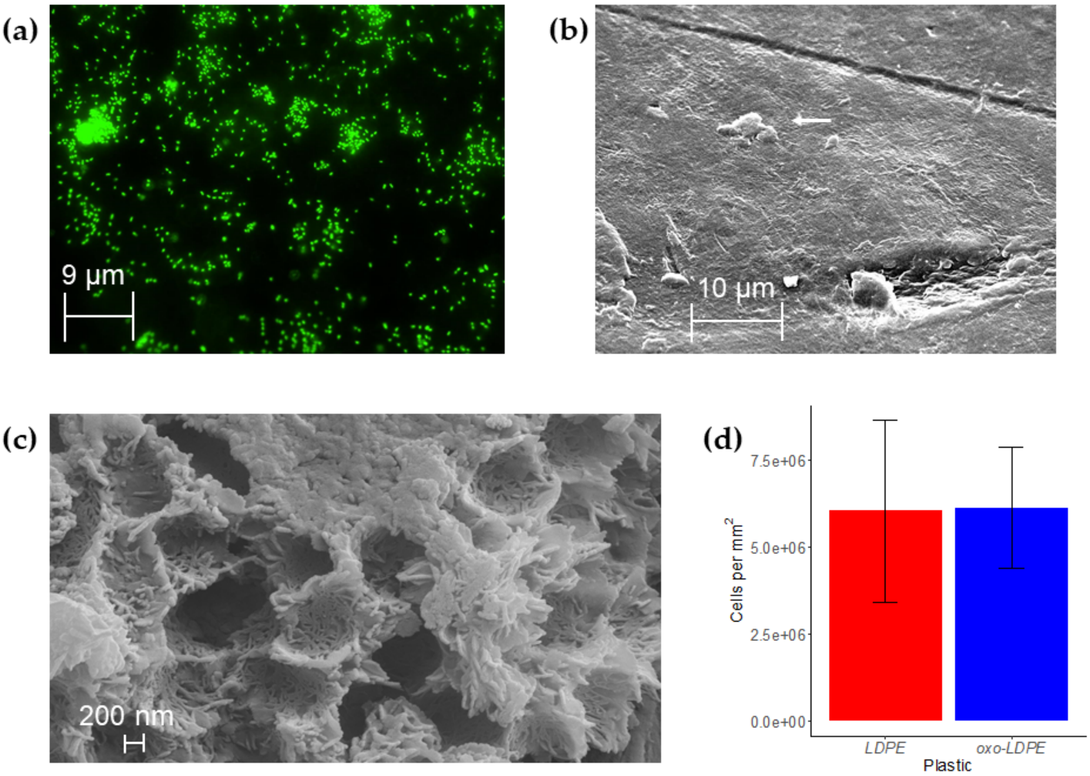

Figure S1: Visualisation of microbial growth on plastic film. (**a**) *R. rhodochrous* was grown on solid media with LDPE as the sole carbon source for 14 days. The film was washed with water and stained with SYBR green (scale bar 9 µm). (**b**) SEM image of *R. rhodochrous* grown on oxo-LDPE. The film was subjected to bleach wash prior to imaging; the arrow indicates the presence of remaining bacteria (scale bar 10 µm). (**c**) SEM image of bacteria grown on oxo-LDPE where the film was acid washed before imaging (scale bar 200 nm). d) *R. rhodochrous* was grown on solid media with LDPE (red) or oxo-LDPE (blue) for 14 days. The film was removed and repeatedly washed with water to remove unbound or dead cells. The cells were stained with SYBR green and counted (n = 3). There was no statistical significance between the colonisation of the two plastics (p = 0.6).

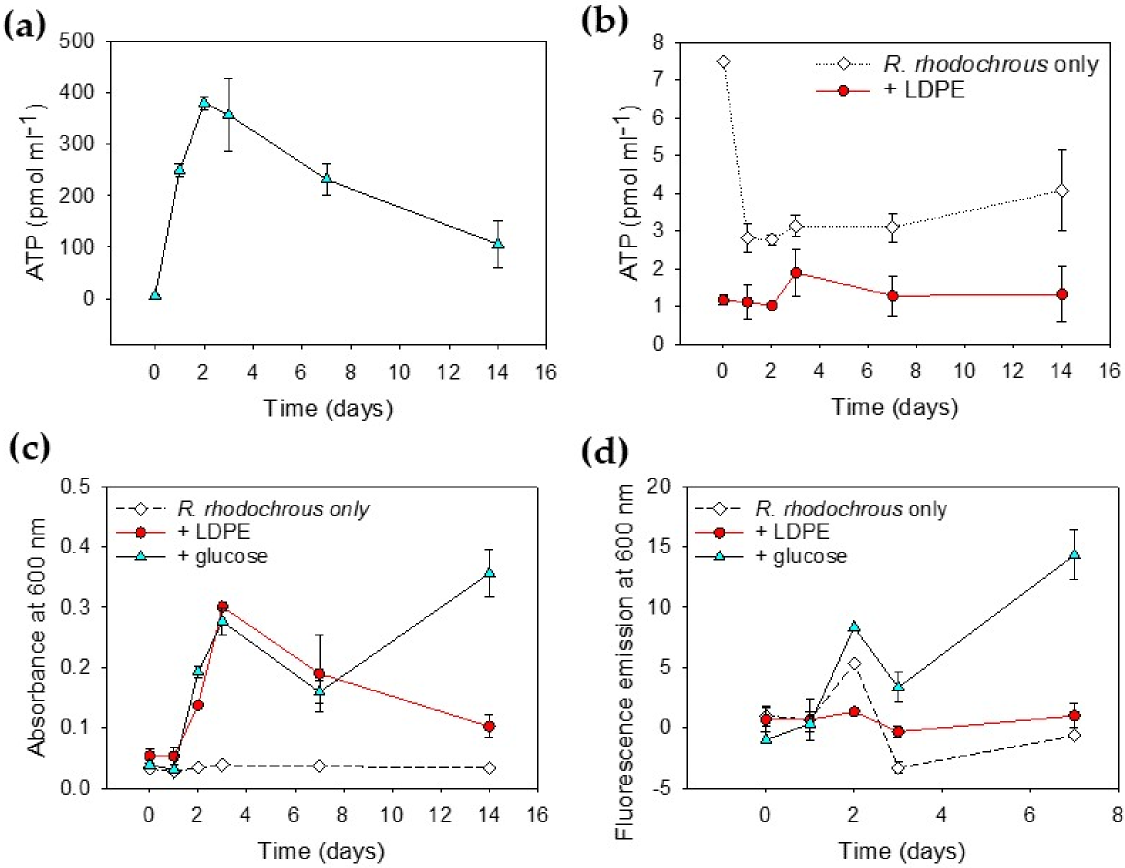

Figure S2: Biochemical methods to measure microbial growth. (**a**) The rate of ATP production measured using the ATP/ADP approach from cells grown in suspension with glucose (cyan triangles) as the sole carbon source (n = 3). (**b**) The rate of ATP production measured using the ATP/ADP approach from *R. rhodochrous* incubated with a polymer film (n = 3). Bacteria grown with no carbon source (open diamonds) died quickly as any residual carbon source was exhausted. There was no evidence of bacterial growth on LDPE (solid circles). No ATP was detected in the initial samples suggesting that the bacteria adhere immediately to the LDPE surface and were not removed sufficiently by the assay buffer. (**c**) Measuring growth by monitoring at 600 nm of *R. rhodochrous* with no carbon source (open diamonds), on LDPE (red circles) or glucose (cyan triangles) (n = 3). d) Measuring growth using the AlamarBlue™ assay of *R. rhodochrous* with no carbon source (open diamonds), on LDPE (red circles) or glucose (cyan triangles) (n = 3).

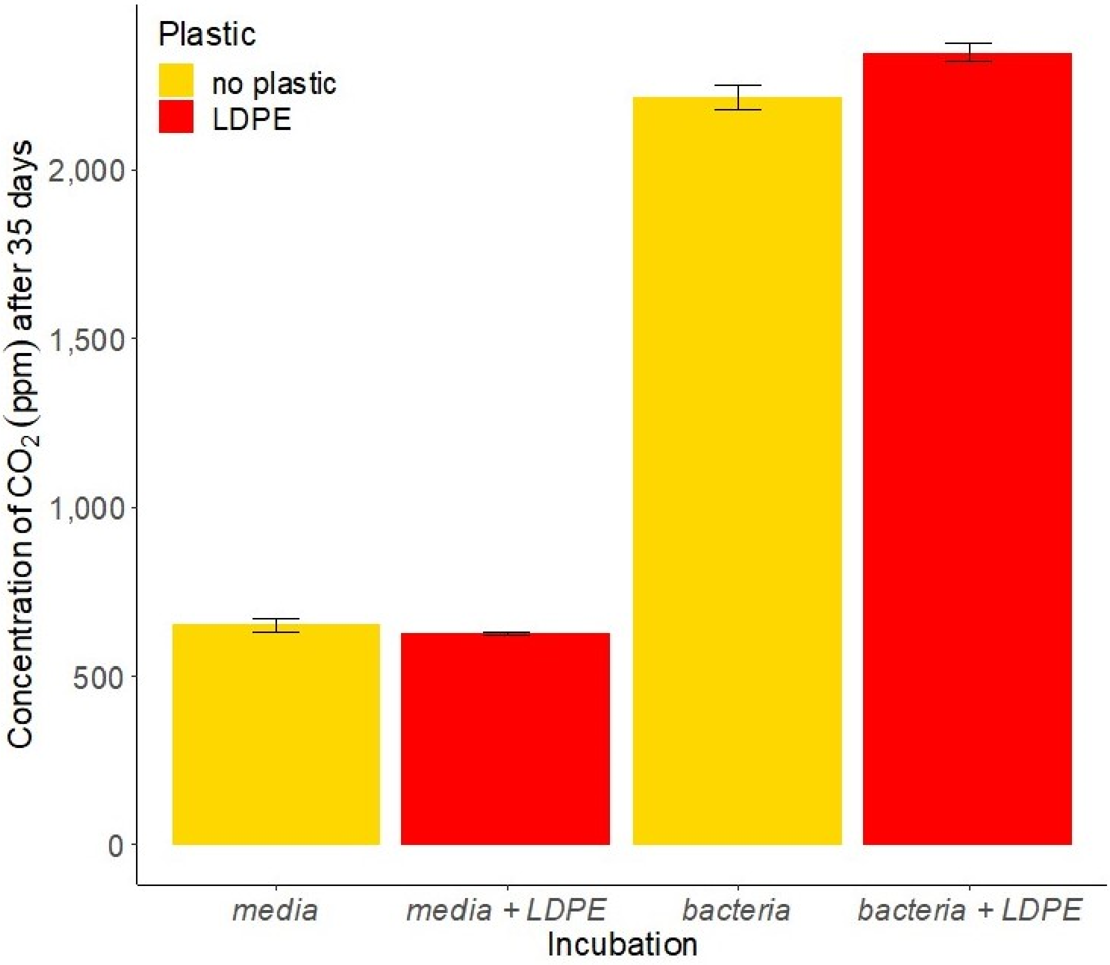

Figure S3: Monitoring the spontaneous release of CO_2 from the control samples after 35 days. There was no significant difference between the concentration of CO_2 in the media with (red) and without (yellow) LDPE time (n = 5, p = 0.8, error bars represent standard error). There was a significant difference between media and *R. rhodochrous* with no plastic (yellow) (n = 5, p < 0.01). Importantly, there was a significant difference between bacteria grown in the presence (red) and absence (yellow) of LDPE (n = 5, p < 0.01). To represent microbial respiration as a result of presence of the polymer, the background of both the polymer alone and bacteria with no carbon source were subtracted.

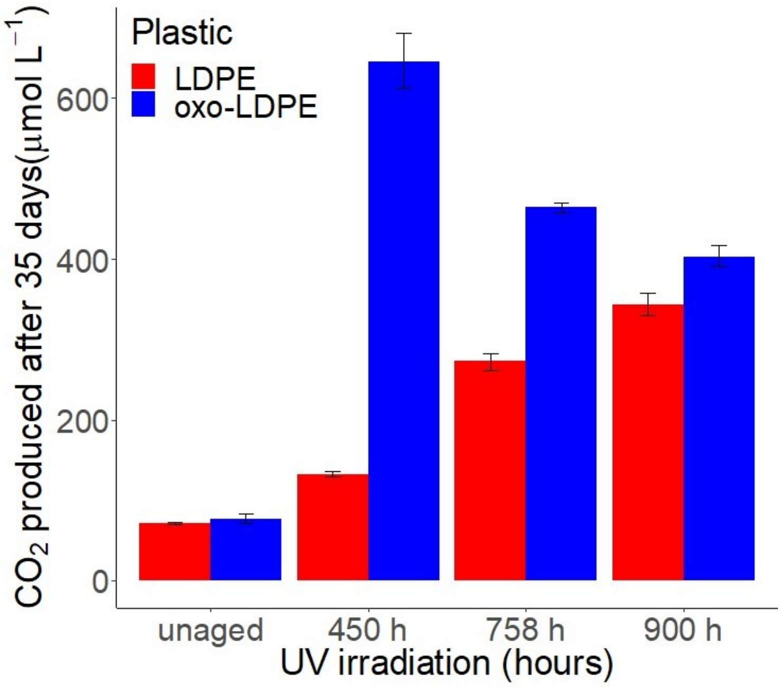

Figure S4: Longer UV exposure increases the biodegradability of LDPE but not oxo-LDPE. CO_2 production after 35 days for *R. rhodochrous* grown on LDPE (red) or oxo-LDPE (blue) that had been exposed to UV for an increasing length of time (n = 5, error bars represent standard error). Note how the degradation of LDPE continues to increase with prolonged exposure to UV whereas the degradation of oxo-LDPE peaks at 450 hours of irradiation.

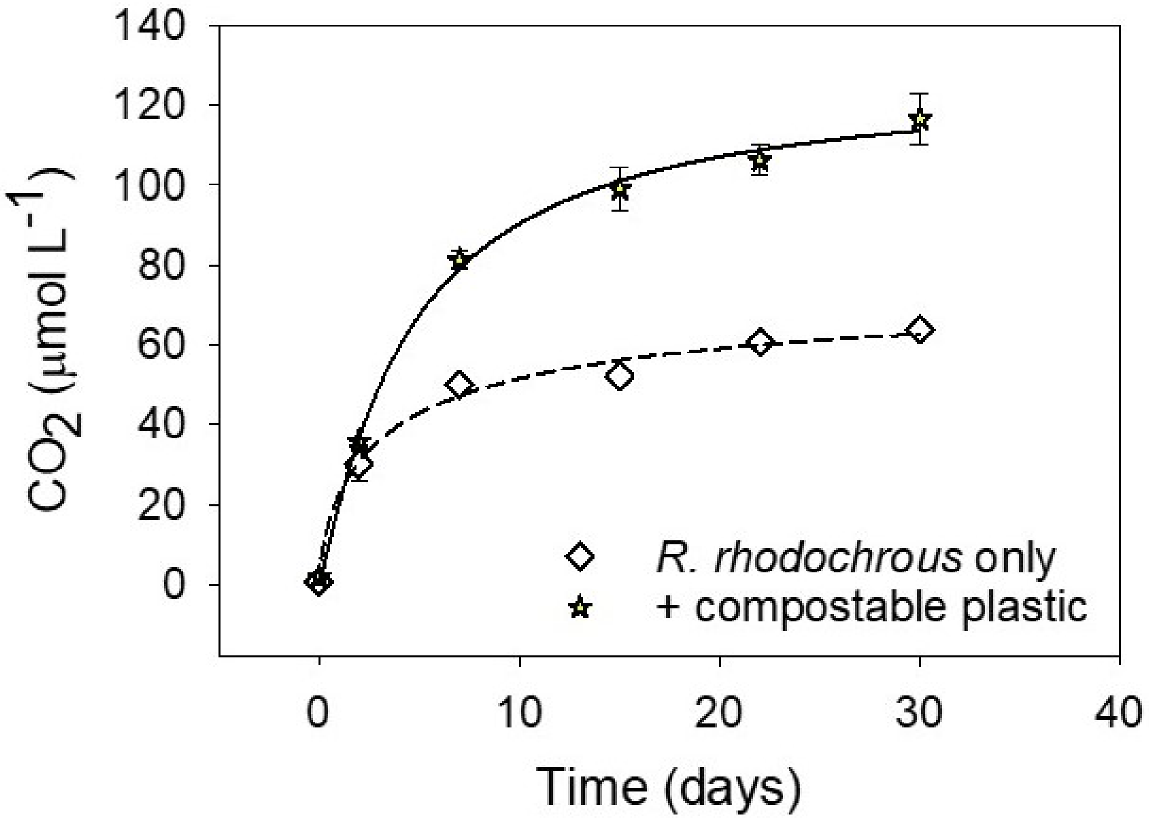

Figure S5: The approach is highly effective at monitoring compostable plastic degradation. *R. rhodochrous* grown with no carbon source (open diamonds/dotted line) or with a sample of a commercially available compostable plastic (yellow star/solid line) and the CO_2 measured over time (n = 3, error bars represent standard error).
